# Validity of transactional analysis and emotional intelligence in training nursing students

**Published:** 2014-10

**Authors:** BRANDI L WHITLEY-HUNTER

**Affiliations:** 1 Masters in health administration, University of La Verne, La Verne, United States

**Keywords:** Emotional intelligence, Transactional analysis, Patient, Communication, Nursing, Students, Curriculum

## Abstract

**Introduction:** Emotional intelligence (EI) is considered a critical component of a nurse’s characteristic trait which is known as a significant predictor of a person’s job performance and life success. Transactional Analysis (TA) plays a fundamental role in nurse-patient communication and managing emotions during difficult dialect with patients. The aim of this review is to discuss the framework of EI and TA, and how the combined theories can be utilized to further educate nurses and enhance the patient’s experience. Exploring the idea of combining EI, TA, and other theories and adding these addendums to the nursing curriculum may advance the empathy and communication skills of nursing students.

**
Methods: **The method used in this review is a literature search using databases, such as Medline, EBSCO, and Google Scholar, etc. to form a critical discussion of this area. Key words such as emotional intelligence, transactional analysis, nursing curriculum, and relating theoretical models were used to identify applicable documents. Four studies involving EI and TA were sampled. A combination of data collection tools, such as lecture series and intervention programs, were used to authenticate the results. Other instruments used were ego state questionnaires, empathy, and five point Likert scales. No study design or type of literature was excluded in healthcare to substantiate the application of EI and TA into the nursing curriculum.

**Results: **Sixteen nurses attended a six-week psycho-education program using communication and empathy scales, and patient satisfaction surveys to improve their empathetic and communication skills. The result of the mean communication score (177.8±20) increased to (198.8±15) after training (p=0.001). The empathy score increased from 25.7±7 to 32.6±6 (p=0.001). The overall result reflects that training can improve emergency nurse’s communication and empathy skills.

**Conclusion: **The data suggests there are under-researched theories with futuristic topics that have value to the nursing community. Suitable evaluation of these theories is vital to nursing education. Implementation and training for nursing students and existing nurses may help shift the culture of medical education ahead by creating a more educated and empathetic work environment.

## Introduction

Patient care has become increasingly multifaceted. The major problem with the attempt to improve patient care is many student nurses put more focus and value towards hands-on-skills while overlooking his or her patient’s feelings. Another contributing factor is that nurse educators often speak of preparing “* safe practitioners*” and “* critical thinkers*, but it is rare to hear faculties speak of preparing a nurse who is “* emotionally intelligent” *(1). EI is defined as monitoring one’s own and other’s feelings and emotions (2).

Lack of broad theoretical education and communication skills is the main factor. Consequently, nursing facilitators face significant pressure to teach students who, upon commencement, are ready to meet the extensive challenges they will encounter in the field. Mastering the technicalities of communication and the ability to express empathy are two of the momentous oppositions students will face. Emotions are vital to the nursing practice and emotional intelligence (EI) is considered an essential characteristic that nurses must have. Lack of this trait can affect the quality of work, clinical decision-making, critical thinking, and the outcome of patient care (2).

Upon assessing the apparent problems of under prepared nursing students, the obvious disconnection lies in the nursing curriculum. The format seems to lack in-depth concentration on areas such as emotional intelligence and techniques to deal with communication roadblocks. EI is on the forefront of health care and is considered a functional universal method. Exploring concepts of EI and transactional analysis in combination with other theoretical framework may bridge the empathetic and communication learning gaps for nursing students. The purpose is to establish the value of EI and TA in the nursing community. The objective is to determine the effect of how early training of those techniques and theories can have a positive impact on how nurses communicate with patients and in turn increase patient satisfaction.

**Defining emotional intelligence**

Emotional intelligence is defined in various ways. Salovey and Mayer (1990) described EI as the subset of social intelligence. EI is to discriminate among oneself and to use this information to guide one's thinking and actions that involve the ability to monitor one’s own and other’s feelings and emotions (2). Salovey and Mayer (1997) created a four-branch model of EI also known as the ability model, which is to “perceive, use, understand, and manage emotions.” To perceive is the most essential element that relates directly to the receptiveness of nonverbal communication; expression of emotion. The “use” of emotion is the capacity to allow emotions to enter and guide the cognitive system to prioritize a creative emotional response towards matters of importance. Emotions convey information; “understanding” emotions is the capability to read the message associated with the actions and determines the best course to manage the overloaded emotional situation. The ability to “manage” is to recognize emotional signals and decipher the information to maintain self-regulation within a person’s personal comfort zone (3). These components are considered the four building blocks for interpersonal and communication skills, which are critical in the nursing field. To identify and manage such actions is an undeniable skill-set.

**Emotional intelligence: An invaluable skill set**

EI is an indispensable skill-set. Practicing nurses have been found to be unprepared to handle emotionally laden situations and complex communication challenges encountered during practice (1). Nurses must have the ability to differentiate between a patient’s and his or her own emotions during communication. At times, a patient’s emotion may be perceived as anger when the true emotion is frustration because of his or her illness. A nurse must have the ability to identify, understand, and manage the situation while controlling herself/himself in order to pinpoint the correct way to respond; EI is an invaluable skill set to do so. The Agency for Healthcare Research Quality in partnership with the Centers for Medicare and Medicaid Services developed the Hospital Consumer Assessment of health care provider’s systems surveys to measure the patients’ experiences with hospital care. The *International Journal of Nursing Practice* (2010) shows data of patients reporting decreased satisfaction with nursing care in hospitals (4).Hospitals must find a way to recover patient dissatisfaction by rebuilding the nurse-patient emotional connection. Emotionally aware nurses are more apt to separate their emotions from distressed patients. Awareness and processing of emotions foster the nurses’ ability to remain focused on the patients' needs and fully engage with clients rather than becoming detached or overwhelmed (5). Unless nurses are able to offer an emotionally intelligent response, the idealized state of a pleased patient may not be achievable.

**Importance of silent communication**

Efficient communication is the foundation to superior nursing skills, which affects the positivity of a patient’s outcome. It is important to understand how people perceive verbal communication during a conversation. Mehrabian’s interpretive theory explains the three types of communication a listener focuses on when an individual is speaking. The three forms of communication are divided into percentages to gain a clear understanding of which category has more prominence when speaking. The interpretive theory reveals;* “words are valued at 7%, delivery (tone, accents on certain words, etc.) 38%, and facial expressions *are most important at *55%” *(6).The interpretive theory supports the significance of understanding nonverbal cues and the correlation between emotions and nonverbal communication. Mehrabian’s theory is parallel with the co-regulation theory in validating nonverbal communication as a critical skill-set in the nursing field.

Fogel is a professor in psychology and has been an active contributor to emotional intelligence research. Fogel’s co-regulation theory supports the importance of nonverbal communication and emotions. Understanding nonverbal communication can be conceived as a practical skill in the nursing field considering many patients only have this option to verbalize his or her needs. Fogel provided a simple explanation of co-regulation; he suggested that a speaker should adjust his/her words or tone of voice based on the perception of the listener's facial expressions or body language, and this may occur on an ongoing basis (7). EI, the co-regulation, and interpretive theory have a correlation with understanding how people communicate, which can be understood by studying transactional analysis.

**Definition of transactional analysis**

Berne (1999) defines transactional analysis (TA) at its simplest level as a method for studying interactions between people. This psychotherapy was developed to help understand the process of communication and what ego state of mind a person may be conversing in while dealing with others. Ego states are examined with two separate models, structural and functional. The structural analysis is divided into three states: parent, adult, and child, which demonstrates how individuals relate to each other (6).The functional parent state is separated into the critical and nurturing parent. Critical ego is based on thoughts and feelings copied from parents or parent figures and the nurturing ego tends to be more protective. The adult ego state relates to thoughts and feelings in the here and now. The child ego state is based on “thoughts and feelings replayed from childhood” (8). Berne (1961) describes the child as having three different ego states with various functions, i.e. the adapted, free, and natural child. The child ego state triggers when a person feels his or her needs are going unmet and a defense mechanism takes over. The “child” is seen as a split between the adapted, natural, and free child. The “adaptive child” is portrayed as the survivor; the “free child” is portrayed as being very energetic, spontaneous, and creative. The “free child wants to do as he or she pleases by taking care of the physical need” (9). When communicating these ego states exists, a person can transfer from one state to another without notice. Nurses must understand how to detect this transference and become properly trained to manage the patient's conversations in a time of illness.

**Correlation between TA and effective communication**

The interpretive and co-regulation theory and transactional analysis inner twine’s; there is a consistent relationship between the three. TA plays an important role in nursing that has gone unnoticed. TA has been successfully used in different professions to develop communication skills such as pharmaceutical lecture series. With a lack of understanding, the various levels of communication and the ability to adjust to fit the mind-set of the patient signals can become crossed and miscommunication can occur. Without the capability to recognize a person’s ego, state of mind and body language, there is a chance of compromising patient care.

**The goal of transactional analysis**

The goal of TA is to have all parties involved converse in the idealized state, which is adult to adult and it remains a challenge. Often a patient in a hospital may experience a feeling of his or her need being unmet and respond in the “free child” ego state of mind. This state can create frustrations for the nurse causing a reaction in the “critical parent” ego state. The feeling of annoyance can create crossed signals, ambiguity, and sarcasm can take over; the patient's needs may go unfulfilled. If the nurse does not have the tools to manage emotions and adjust to fit the mindset of the patient, the idealized state is lost. This model will address the how, what, and why the deviation from the idealized state exists from self or the patient. This research has been an attempt to assess the effect of transactional analysis combined with emotional intelligence as a tool used in the nursing curriculum to educate nursing students early on. The expectation is to advance empathy and communication skills for student and veteran nurses, which in turn can create progress with patient outcomes and personal satisfaction. 

## Methods

The method used in this review is a literature research involving subjective data on emotional intelligence and transactional analysis. The collection of samples from the literature involves qualitative and quantitative methods. Qualitative tools such as semi-structured and cognitive interviews, lecture series, and unstructured observation practice were used to corroborate the data. Literature research involving quantitative methods were used such as convenience sampling, Mayer-Salovey-Caruso Emotional Intelligence Test (MSCEIT) and 360 test, student and faculty information forms, and ego states questionnaires to authenticate the outcome of the statistical results. 

The methodology used in this review was inspired using Eric Berne, Daniel Goleman, Peter Salovey, and John Mayer’s theoretical work as a primary guide. The work of Alan Fogel’s and Mehrabian were utilized to evaluate the possibilities of combining the theories to improve the current nursing curriculum. The sampling of literature is in hopes to motivate additional exploratory research to determine if incorporating EI and TA into the current curriculum can increase the knowledge base of student nurses. The idea is the addendum could develop a heightened level of empathy and communication skills to benefit nursing students, improve patient outcomes, and increase satisfaction. 

The databases used in this literature search were the Agency for Healthcare Research and Quality (AHRQ), the National Library of Medicine, National Center for Biotechnology Information (NCBI), National Institutes of health, EBSCO, and Google Scholar to research subject related literature. Various search terms were used to the compile the data such as, emotional intelligence, emotional intelligence in nursing, transactional analysis, emotions, patient communication, nursing curriculum, empathy, co-regulation, nonverbal communication, and patient satisfaction. The literature will address where research has been and where expansion is needed for progression in the nursing field. 

## Results

**Lack of communication creates errors and redundancy**

Communication is an obvious foundation to superior nursing care; the U.S. benchmark average of patient communication with nurses is 78%. The summary of Hospital Consumer Assessment of Healthcare Providers and Systems (HCAHPS) survey results from April 2012 to March 2013 reports large states such as New York and California are under average with 4%. Other states, such as Louisiana seem to be trending in the right direction of nurse patient communication; their reported results are 83%. Although the benchmark average is a fair start, there is still a need for first-rate communication results in this area (10).

Since the 2006 report, the Joint Commission Sentinel Event Advisory Group has implemented the National Patient Safety Goals, with revision in 2011, that focuses on improved communication effectiveness among nurse caregivers. The cause of this implementation is more than 4800 reported serious and preventable events that cite ineffective communication as a frequent contributor to medical errors. The 2011 goals that are expected to be executed by nursing caregivers are timely, accurate, and completely unambiguous communication that is comprehensively understood by the recipient. The expectation is that the improvements will reduce communication and medical errors and result in improved patient safety. Effective communication and handoff responsibility is an essential component of nursing practice and clinical education. It is critical to cultivate a culture of safety in nursing students with effective routines that will continue throughout their careers. Careful attention to detail “must” be stressed as a fundamental aspect in nursing care. Nursing students must adamantly be trained in the area of “minuscule detail” and learn how to communicate information efficiently at the time of transferring care to reduce error (11).

The Joint Commission Center for Transforming Healthcare has taken some steps to reduce the error rate by developing the targeting solutions tool (TST) and executing the hand- off communication program. This program has reduced 50% in communication errors between the patient and care giving team (12).This result is a clear indication that communication is a fixable and attainable goal. The outcome is an indication that health care education for nurses should focus on in-depth communication and empathy classes from the beginning. Leaders in health care, such as nurses, must understand the consequences of communication behavior. Communication involves more than spoken words; the empathetic demeanor, finesse and delivery of those words have a direct effect on patient care. 89% of communication is considered nonverbal (6). Tackling these issues from the start by using TA techniques and EI theoretical framework can reduce the redundancy for nursing students entering the health care field.

**A nurses’ perception determines their response**

A study based on “how nurses respond to patients” by Sheldon and Ellington (2008) surrounding Orlando’s theory of how nurses process patients behavior was implemented in 2006. The process illustrates how nurses generate a perception, thought, feeling, and take action based on the patient’s behavior. The data was gathered through cognitive interviews combined with convenience sampling of five nurses utilizing the crick and dodge model of social processing. The social information model describes the sequential steps in the cognitive process used to respond to social cues that may be useful in explaining the nursing process. The outcome of the investigation is that nursing communication affects patient outcomes such as anxiety, adherence to treatments, and satisfaction with care. The conclusion is models of social information processing enhance the understanding of the process of how nurses respond to patients and construct further development for nursing theories (13). This study illustrates the importance of nonverbal communication in nursing. It also supports the notation that the combined theories of co-regulation, TA, and EI may be a useful addendum to the nursing curriculum.

**Lecture series involving the TA method**

The American Council of Pharmaceutical Education’s Accreditation Standards and Guidelines, and the American Association of Colleges of Pharmacy’s (AACP), and Center for the Advancement of Pharmaceutical Education (CAPE) outcomes include communications skills as assessment and outcome criteria for pharmaceutical students. A 10-week course using the TA method was developed to help pharmaceutical students communicate more effectively with patients. During the lecture, students were asked to take a personality assessment, apply the learned techniques, and report their experiences. The course was devoted to developing a knowledge base in effective communication that included practicing patient communication through case scenarios. A case scenario is a similar method that is used in nursing education. The focal point was to use the lecture series as part of the educational process of patient communication. Students are presented with the concept of TA and two methods of personality assessments. The objective of the segment and the patient counseling communications course is to help students understand psychological factors that may affect patient communication (14).

**TA method works**

The result of the lecture series is students could demonstrate and apply the techniques with an understanding of the psychological factors that may influence patient communication. Additional findings support the students’ capability to engage patients based on adult-to-adult interaction cues. Students also gained a greater awareness of transactional analysis and personality assessment by applying these concepts (14). The similarities between nurses and pharmacists are quite astonishing concerning communication with patients in the hospital. It is evident from this lecture series that the ability to understand and use TA will help pharmaceutical and nursing students effectively communicate with patients in diverse settings.

**Managing diversity with TA**

Managing diversity is a factor in the lecture series that has a correlation with TA and directly affects patient communication. The health care community is extremely multi-cultural. Patients have different personality types and understanding how to appreciate diversity and manage communication can be honed using TA techniques. Recognizing that all patients do not respond similarly because of cultural dissimilarities and personality traits improves the students’ tolerance for these differences. 

**TA trending in health care studies**

The use of TA has taken a positive trend in a variety of areas in health care. The method was used in a lecture series as the research design to train pharmacists how to identify ego states and make adjustments to communicate effectively with patients. This method is also being used in clinical settings**. **An experimental double blinded study using the TA method was used in a hospital setting to evaluate the effect TA education can have on nurses to improve the patients‏‏' satisfaction. The study involved real hospitalized patients and non-medical students were selected to participate as standardized patients. Before each hospitalization, there was a session with related specialists in the wards to help standardized patients play their role successfully without causing any disbelief (15).

Participants were initially assigned to an intervention or control group and asked to complete a patient satisfaction questionnaire regarding nurse’s communication skills. The instrument used in the study was a 5-point Likert scale consisting of 18 questions regarding how nurses communicate. The value of the scale was 1 (strongly disagree) and 5 (strongly agree). The mean scores for all nurses were calculated (69) and 25 nurses were divided into two groups; 1-nurses with scores higher than mean and 2-nurses with scores lower than mean. The nurses were randomly divided into two subgroups; 13 in the case and 12 in control groups. Participants in the case group received a brief training about TA. Real hospitalized patients and standardized patients’satisfaction was followed up after one week, one month, and three months. In spite of a brief educational intervention program, the outcome of the study was positive in the short term with immense long-term potential. The positive effect of the study began to diminish over the course of one to three months, which gives an indication that repeated intervals of the TA program may bring long-term success. Overall, the study demonstrated the validity of TA as a universal communication method that is useful in the nursing field and enhances patient satisfaction (15).

**Can empathy be taught?**

Over the last decade, there have been debates regarding EI being a trait someone is born with or if the connection is to one’s IQ, or can empathy be taught and learned. Theorists seem to have a difference of opinion, but the agreement is EI can add value to the success of one’s life. Killian (2012) implied “emotional competencies have been found to contribute to outcomes such as life satisfaction, general health, work, academic performance, and leadership potential” (16).

In the (2006) MSCEIT report, Salovey and Mayer’s (1997) research on the subject concluded that EI is an actual intelligence; it can be measured through an ability test. This test, known as the MSCEIT, is based on the ability model of EI and consists of perceiving and identifying emotions, assimilating and using emotions, understanding emotions and managing emotions (17). In Goleman’s (1995) book *Emotional Intelligence*, he declared that EI is a more important predictor of job performance and life success than the Intelligence Quotient (IQ) (18). Goleman’s (1995) mixed model of emotional intelligence, which contrasts with Salovey and Mayer’s ability model comprises of five different elements within the construct of EI. The mixed model consists of “self-awareness, self-regulation, motivation, empathy, and social skill” (Goleman, 2004). He posits that individuals are born with a general emotional intelligence that determines their potential for learning emotional competencies (19).

Emotional competencies are not innate talents, but rather learned capabilities that must be worked on and developed to achieve outstanding performance. In the Harvard business review article, Goleman (2004) believes in both; an individual can be born with EI and learn to develop the trait over time. Research and practice clearly demonstrates that EI can be learned; like maturity, EI increases with age.” The outcome of this measurement reveals that it is “this ability that differentiates one’s exceptional capability from mediocre skill and achievement” (19).

**Emotional intelligence training produces results**

Emotional intelligence training for nurses of all levels can bring positive results because of patient interaction. A psycho-educational study was implemented in 2011, consisting of theoretical education on empathy and communication. The study is comprised of sixteen emergency nurses attending a six-week psycho-educational program intended to improve their empathetic and communication skills. The first three sessions of the program consisted of theories focusing on empathy and communication. Additional sessions covered awareness, active communication, and empathic skills on a cognitive behavioral basis using discussion, role play, and homework within an interactive group. The effects of the program were assessed using communication skills and empathy scales, and a patient satisfaction survey. The outcome was documented and reflected by a reduction in the number of undesirable events between nurses and patients in the emergency department. The result of the mean communication score (177.8±20) increased to (198.8±15) after training (p=0.001). The empathy score increased from 25.7±7 to 32.6±6 (p=0.001). The overall result reflects training can improve the emergency nurse’s communication and empathy skills. EI and communication education can reduce complaints and undesirable interactions between nurse and patient (20).

In the article *Can Empathy be Taught*, a research team of 99 resident physicians from six different specialties were enrolled in the study and their patients were asked to rate the physicians’ empathy before the training period. The primary outcome from the study was the CARE measure. The focus was rating their doctors on various items like “really listening,” “showing care,” “compassion,” “answering questions,” and “explaining things clearly”. The findings from the research imply that without specific training physicians’ empathy declines. Ninety-five percent of the physicians found the training to have a positive impact; the training was interesting, helpful, and likely to be used as a new tool in practice. The relevant finding from the study is that empathy can be taught. Medical professionals learned they should be aware of the underlying vulnerability of their patients' surface behaviors and be able to manage their own emotions (21).

**Validity of non-traditional intelligence**

Empathy is a foundation skill that makes up most of what constitutes emotional intelligence. Nursing abilities that depend on “*nontraditional intelligence”* are not typically taught during the educational process. It is apparent that much of nursing depends on this intelligence, which is crucial for efficient patient care (22). Patients appreciate an empathetic attitude and put more value on a nurse with a compassionate attitude. Emotional intelligence is now being viewed as a technical skill in health care (23). The selection process and the possibility of identifying nursing recruits who are high in EI can be accomplished through MSCEIT test as proven through scientific research. The MSCEIT test measures individual abilities of perception of emotion, knowledge of emotions, and management of emotions (17). MSCEIT is executed by specific tasks such as examining pictures, drawing parallels between emotion and physical sensation, and describing how emotions change and the way people manage their own and others’ emotions (16). Using this test as a selection tool in hospitals to hire nurses with the desired characteristics to care for patients may change the patient outcomes. This tool may serve as an indicator to determine the level of empathy a student possesses upon entrance and the progress made during the program. To accomplish this task, coordination is required with nursing schools to incorporate EI, TA, and other theoretical models within the curriculum to enhance futuristic nursing. Health care corporations have to take initiative and support the changes by taking action and implementing a screening process to ensure successful hires.

**Managing emotions can prevent burnout**

Cordier et al. (2011)**provides a relevant example of how nurses frequently have difficulty accepting their own anger at patients. During patient care, nurses have a tendency to experience negative feelings such as hostility, which can be directed towards the patient. In that moment, the nurse may not be able to manage his/her feelings appropriately; this can compromise patient care. Understanding an emotion involves appreciating its depth, diversity, and complexity (22). Once a nurse correctly identifies, understands, and uses emotion to facilitate reasoning, there is a greater ability to manage emotional situations effectively. Mastering these abilities can increase the level of communication and in turn improve the patient’s satisfaction. EI research in nursing suggests there is “*vast potential*” for this concept to improve nursing performance, prevent burnout, and recuperate retention.

## Discussion

The old theory in nursing education has often been viewed as an essentialist education with an emphasis on enhancing the training process rather than educational development. Essentialist education by its very nature molds the student (24). McQueen (2004) implied that early education encouraged nurses to conceal their emotions as a professional barrier; this conferred some protection from the emotional concerns of patients. The early curriculum was considered suitable for a traditional nursing syllabus, which was administered by the General Nursing Council (25). The current research implies that the curriculum falls short of emphasis on critical factors such as empathy and communication. The implication of facilitators embodying the value of empathy in the classroom is insufficient. Not only is it critical to train new students, it is imperative that the American Nursing Association starts with the facilitators. If facilitators are not trained in EI and TA, nursing students experience insufficient development of emotional competencies. These essentials can assist a nurse in achieving balance in providing the emotional care necessary for the patient. The students’ first contact is with the instructor. Learning how to express empathy should be conveyed through the instructor to the student with an addendum to the curriculum. The change requires professors to become intimate with their emotions and facilitate learning from a position of self-knowledge. Professors who find themselves emotionally removed from their working environment can find it challenging to put themselves in a caring situation with students when training (24).

Wheeler and Barret (1994) have reported low empathy levels in nursing instructors and additional studies by MacKaey et al. (1990) and Reynolds (1998) indicate levels remain low in many areas of nursing practice. This “raises concern whether the nursing field has sufficient role models to enable students to develop a higher level of therapeutic skills” (26). The recommendation by Cadman and Brewer (2001) is to recruit students who demonstrate potential or already possess the skill-set. The result of the case is to increase the recognition of EI’s contribution to improving patient outcomes and clinical leadership (26). “This framework may be one of the most vital theoretical models that can help shift the culture of medical education ahead by creating a more empathetic work environment” (27).

Creating a more caring work environment starts with the curriculum and facilitator. An addendum to the nursing curricula is a start, but emotional intelligence needs to be “firmly placed at the core of the educational program”. It is fundamental that the rational and emotional dimensions are incorporated into the intellectual functioning of the curriculum, which can enhance health care practices (26). Despite the overwhelming evidence the debate remains if EI and TA are relevant to the health care community**. **A quote in an article about emotional intelligence by Freedman et al. suggests “emotional intelligence is responsible for about 80% of the success in our lives”. “It defines how and what we learn; think, and react to what happens in our lives” (28). EI training can re-direct a nurse’s emotional reaction. Students can learn empathy training and acquire the necessary knowledge by the use of reasoning and intuition. “Compassion can be learned through acknowledgement of how hard it is to be sick or feel pain by utilizing one’s emotional intuition” (21). Teaching nursing students to make a human connection can define integrity and moral standards can surface and move them in the right direction. When nurses are kindhearted and display empathy, a patient often feels he or she is receiving a greater level of care.

The *Journal of Clinical Nursing*: the article entitled “professional tears” (2011) provides evidence to support the importance of how EI education provides preparation for the nursing field. Emergency nurses in hospitals must have a deep sense of awareness and develop expertise in end-of-life care giving. A nurse can this accomplish through “three stages of development: (a) investment of self in the nurse–patient relationship, (b) managing emotional labor, and (c) development of emotional intelligence”. The methods used in this study consisted of unstructured observation practice and semi-structured interviews. The findings of this research indicate EI can help nurses manage their emotions when dealing with dying patients, while investing into their therapeutic-self (29). The performance level of nurses has been positively correlated with EI. This model can assist in directing the patient's response and redirecting the nurse's emotional reaction (4).

## Conclusion

The majority of studies that have been conducted focused on EI and TA in nursing separately. Strides in developing innovative curriculum have been attempted without progress. The course work and ethical experiences should provide the graduates with the knowledge and skills in empathy to display in nursing by using the appropriate theoretical models and ethical framework (30, 31). The exploratory research falls short in the initiation of combining the work of Berne, Fogel, and Mehrabian to enhance the full circle of nursing. Based on scientific research, the theoretical framework from the mentioned theorists may bring further development to the nursing curriculum and enhance the patient’s satisfaction.
